# Associations of accelerometer-measured physical activity and sedentary time with renal function and chronic kidney disease: a national population-based study

**DOI:** 10.3389/fendo.2024.1403998

**Published:** 2024-06-17

**Authors:** Xiangying Suo, Yanhua Liu, Adwoa Nyantakyiwaa Amoah, Yacong Bo, Quanjun Lyu

**Affiliations:** ^1^ Department of Epidemiology and Health Statistics, School of Public Health of Zhengzhou University, Zhengzhou, China; ^2^ Department of Nutrition, The First Affiliated Hospital of Zhengzhou University, Zhengzhou, China; ^3^ Department of Nutrition and Food Hygiene, School of Public Health of Zhengzhou University, Zhengzhou, China; ^4^ NHC Key Laboratory of Birth Defects Prevention, Henan Key Laboratory of Population Defects Prevention, Zhengzhou, China

**Keywords:** chronic kidney disease, physical activity, sedentary behavior, accelerometers, estimated glomerular filtration rate, isotemporal substitution model

## Abstract

**Introduction:**

There is limited information about the relationship between physical activity (PA) and sedentary behaviors in chronic kidney disease (CKD). Therefore, this study aims to explore the associations of accelerometer-measured PA and sedentary behaviors with CKD.

**Methods:**

A cross-sectional study was conducted using data from the National Health and Nutrition Examination Survey in the 2003–2004 and 2005–2006 survey cycles. A uniaxial accelerometer measured physical activity (PA) and sedentary time (ST). The associations of PA and ST with estimated glomerular filtration rate (eGFR) and odds of CKD adopted the generalized linear regression, multivariable logistic regression, and isotemporal substitution models.

**Results:**

A total of 5,990 adults with 605 CKD patients were included in this study. Compared with the individuals in the first quartile group, participants in the fourth quartile of low-intensity physical activity (LIPA), moderate to vigorous physical activity (MVPA), and ST were associated with 52% (35%, 65%) and 42% (14%, 62%) lower odds of CKD and 64% (17%, 131%) higher odds of CKD, respectively. Substituting 30 min/day of ST with equivalent LIPA/MVPA contributed to risk reduction in CKD.

**Discussion:**

The findings suggest that increased LIPA and MVPA and reduced ST were associated with a lower risk of CKD and that replacing ST with LIPA may decrease the risk of CKD.

## Background

Chronic kidney disease (CKD), characterized as the progressive deterioration in renal function, was the 11th leading cause of death globally in 2019 (1). The U.S. Centers for Disease Control and Prevention found that approximately 37 million U.S. adults (representing 15% of the U.S. population) are estimated to have CKD (2). Increasing lines of evidence have demonstrated that patients with CKD are at higher risk of bone disorders and fractures, cognitive decline, infection, anemia, poor quality of life, and all-cause and cardiovascular mortality (3–7). Many modifiable risk factors, including unhealthy lifestyles and toxic environmental factors, may contribute to the incidence and progression of CKD (8–10).

Physical activity (PA) is regarded as an essential intervention for the prevention of most chronic diseases, such as cardiovascular disease, metabolic syndrome, and cancer, which share common risk factors with CKD (11–13). However, there are conflicting findings about the relationship between PA and CKD, which may be ascribed to different measures of PA. Most of them suggested that higher PA is beneficial for renal function (12, 14, 15) while some studies showed no relationship (16, 17).

It has been increasingly recognized that sedentary behaviors, independent of PA, were modifiable risk factors for several health outcomes, including functional limitations (18, 19), metabolic risk factors (20), and all-cause and cause-specific mortality (21, 22). There is limited evidence on the joint associations of accelerometer-measured PA and sedentary time (ST) with the risk of CKD (14, 23). In addition, most of them were based on self-reported questionnaires (14, 24, 25), which may lead to the underestimated health effect of ST (26, 27). To the best of our knowledge, only the Framingham Offspring Study (FOS) has investigated the relationship between accelerometer-measured PA, ST, and CKD (15). However, that study mainly targeted on an elder population of white individuals of European ancestry. A cross-sectional study highlights that replacing sedentary behavior with PA may benefit renal health in older adults (28). However, the potential interrelation between ST and PA with CKD/renal function in adults is not well understood. Therefore, we conducted a national representative cross-sectional study of 5,990 adults from the National Health and Nutrition Examination Survey (NHANES) to investigate the relationship between objectively measured PA and ST with the prevalence of CKD.

## Methods

### Study population

The participants of this study were from the NHANES, which is a national survey conducted by the U.S. National Center for Health Statistics (NCHS). In brief, the NCHS conducted a series of in-home interviews and medical examinations bi-annually in a mobile examination center (MEC) (29). During each survey cycle, a complex, stratified, and multi-stage sampling method was adopted to select a representative population in the United States. Information on lifestyle factors and health and nutritional status was collected for each participant. All the protocols were approved by the NCHS Research Ethics Review Board and written informed consent was obtained from all participants.

**Figure 1:**
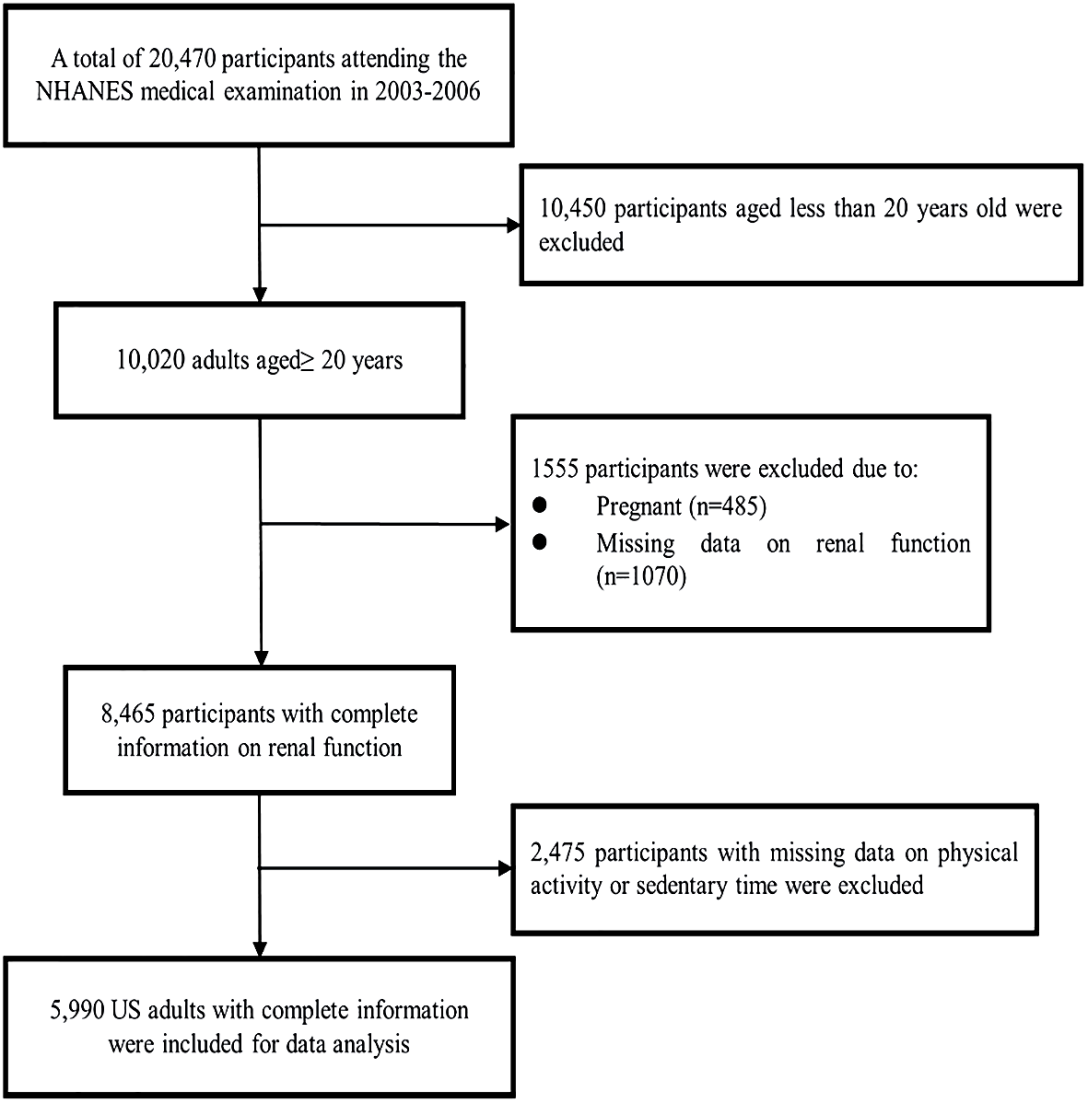
Flowchart of participant selection.

In this study, data were collected from adults aged 20–85 years who were enrolled in the 2003–2004 and 2005–2006 survey cycles. **Figure 1** shows the procedure of participant selection. Briefly, 20,470 U.S. residents attending the NHANES medical examination were recruited during 2003 and 2006. Among them, 10,450 participants were excluded due to age less than 20 years old, and 485 pregnant participants were excluded as pregnancy may bias the analysis. Subsequently, 1,070 participants with missing information on renal function were excluded. Furthermore, 2,475 participants were excluded due to absent data about sedentary behaviors. Finally, 5,990 adults were included in the main analysis.

### Measurement of PA and sedentary time

The measurement method of PA and ST has been described in previous publications (22, 30). Briefly, participants were instructed to wear a uniaxial accelerometer (AM-7164, Acti-Graph) on their waist for 7 days, and to remove it during sleeping, bathing, and swimming. The bodily movement was recorded as 1-min epochs and saved as an activity count (AC). AC values were used to identify sedentary behavior and non-wear periods. Non-wearing time was defined intervals of 0 cpm for at least 60 consecutive minutes, with up to 2 allowable minutes of some limited movements (AC < 50 cpm). Days with at least 10 h of wearing time without excessively high values (>20,000 cpm) were considered valid. In order to evaluate the long-term pattern of PA, we only included participants wearing the accelerometer for at least 4 valid days, including at least one weekend day, in the analysis. An ST was defined as wearing time with AC < 100. A low-intensity physical activity (LIPA) was defined as an AC value of 100–760. A moderate to vigorous physical activity (MVPA) was defined as an AC value of ≥760. In order to evaluate PA and ST, we included these three aforementioned variables of interest in this study: (1) total duration of ST (hours), (2) LIPA, and (3) MVPA.

### Definition of renal function and CKD

The health outcomes of interest for this study were renal function [i.e., estimated glomerular filtration rate (eGFR)] and the prevalence of CKD. The modification of diet in the renal disease (MDRD) study was used to calculate the eGFR as follows (31):

eGFR = 186.3 × (serum creatinine) ^−1.154^ × age^−0.203^ × (0.742 for women).

in which the concentration of serum creatinine was treated as mg/dL. Participants were considered to have CKD if their eGFR <60 mL/min/1.73 m^2^ (31).

### Covariates

All participants were asked to complete a standard questionnaire by trained interviewers to collect detailed information on potential confounders, including participants’ demographic characteristics [sex (male or female), age (years), educational level (<high school, high school graduate or general equivalency diploma, some college or associate degree, and ≥bachelor’s degree), and ethnicity (Hispanic, non-Hispanic white, non-Hispanic black, and race—including multi-racial)], lifestyle factors [smoking (never, former, or current) and alcohol use (nondrinker, low-to-moderate drinker, or heavy drinker)], and ratio of family income to poverty (≤1, 1–3, or >3). Body mass index [BMI, kg/m^2^] was calculated as weight in kilograms divided by square of height in meters.

### Statistical analysis

We used numbers (percentages) to depict categorical variables and means (standard deviations) to describe continuous variables. The multiple imputation was used to impute the missing covariate values (except for drinking, all other covariates had <5% of the missing values) by using the R package “mice (version 3.16.0)” (32).

To investigate the relationship between PA and ST and renal function (i.e., eGFR), the generalized linear model was adopted by the R package “stats” (version 4.2.2). The eGFR was naturally log-transformed to normalize the data and analysis and the original scale was then transformed back for presentation. The percentage difference of eGFR was reported as effect estimates, with individuals in the first quartile of the sedentary behaviors as the reference. The percentage difference was calculated by using:


$$\left(e^{\text{β}}-1\right)\;\times \;100\%$$

where β is the corresponding coefficient. The adjusted models were introduced by incrementally adding covariates: Model 1 was crude model; Model 2 was adjusted for age, sex, educational level, and BMI; Model 3 was further adjusted for smoking, alcohol use, and ratio of family income to poverty.

We adopted the multivariable logistic regression model to evaluate the associations between PA and ST and the odds of CKD by the R package “stats (4.2.2)”. These three aforementioned models were also introduced to estimate the odds ratio (OR) and corresponding 95% confidence interval (CI).

To evaluate the joint associations of PA and ST with renal function and CKD, we also categorized the participants into low and high ST, LIPA, and MVPA, respectively. First, we classified the participants into 4 mutually exclusive groups according to the categories of LIPA and ST: high ST and low LIPA (reference); high ST and high LIPA; low ST and low LIPA; and low ST and high LIPA. Second, we categorized these participants into four mutually exclusive groups according to the categories of MVPA and ST: high ST and low MVPA; high ST and high MVPA; low ST and low MVPA; and low ST and high MVPA. The effect estimates (i.e., change% and OR) for each joint exposure group associated with eGFR/CKD were evaluated using the aforementioned Model 3.

The isotemporal substitution model was adopted to evaluate the interrelation between ST and PA with CKD/renal function by the R package ‘‘compositions (version 2.0–8)” (33). For example, when we examined the effect of replacing ST with moderate to vigorous activity, we dropped ST from the model but retained light activity, moderate to vigorous activity, total accelerometer wear time, and other covariates in the mode.

Stratified analyses were conducted to evaluate the potential effect modification by sex (men or women), BMI (<25 kg/m^2^ or ≥25 kg/m^2^), and ethnicity (white, black, or others). We examined each potential modifier separately by adding a multiplicative interaction term (i.e., continuous ST/PA* potential modifier).

All statistical analyses were conducted using R 4.0.2. (R Core Team, Vienna, Austria), and two-sided *p*-values <0.05 were considered statistically significant.

## Results

### Characteristics of participants

The general characteristics of included study participants are presented in **Table 1**. Among the 5,990 adults included, 605 participants were diagnosed as CKD. In comparison to participants without CKD, those with CKD were older, were more likely to be non-Hispanic white, have lower educational levels, were more likely to be former smokers, and were less likely to be heavy drinkers.

**Table 1:** Characteristics of study participants based on the presence of CKD, the United States, 2003–2004 and 2005–2006 survey cycles.

Variables	Total (*n* = 5,990)	Non-CKD (*n* = 5,385)	CKD (*n* = 605)
**Age, years**	52.90 (18.30)	50.63 (17.57)	73.01 (10.73)
Sex			
Female	3,093 (51.64%)	2,825 (52.46%)	268 (44.30%)
Male	2,897 (48.36%)	2,560 (47.54%)	337 (55.70%)
Race			
Hispanic	1,392 (23.24%)	1,320 (24.51%)	72 (11.90%)
Non-Hispanic White	3,243 (54.14%)	2,800 (52.00%)	443 (73.22%)
Non-Hispanic Black	1,118 (18.66%)	1,053 (19.55%)	65 (10.74%)
Other Race— Including Multi-Racial	237 (3.96%)	212 (3.94%)	25 (4.13%)
Education level			
<High school	1,635 (27.30%)	1,429 (26.54%)	206 (34.05%)
High school graduate or general equivalency diploma	1,472 (24.57%)	1,299 (24.12%)	173 (28.60%)
Some college or associate degree	1,657 (27.66%)	1,531 (28.43%)	126 (20.83%)
≥Bachelor’s degree	1,226 (20.47%)	1,126 (20.91%)	100 (16.53%)
Drinking			
Never	1,647 (27.50%)	1,375 (25.53%)	272 (44.96%)
Low to moderate	1,413 (23.59%)	1,267 (23.53%)	146 (24.13%)
Heavy	2,930 (48.91%)	2,743 (50.94%)	187 (30.91%)
Smoking			
Never	1,169 (19.52%)	1,119 (20.78%)	50 (8.26%)
Former	1,749 (29.20%)	1,497 (27.80%)	252 (41.65%)
Current	3,072 (51.29%)	2,769 (51.42%)	303 (50.08%)
Ratio of family income to poverty			
≤1	910 (15.19%)	843 (15.65%)	67 (11.07%)
1–3	2,569 (42.89%)	2,232 (41.45%)	337 (55.70%)
>3	2,511 (41.92%)	2,310 (42.90%)	201 (33.2%)
**BMI, kg/m^2^**	28.40 (6.20)	28.38 (6.28)	28.7 (5.72)

CKD, chronic kidney disease; BMI, body mass index.

### Associations between physical activity, sedentary time, and eGFR/CKD

The associations of PA, ST, and eGFR are shown in **Table 2**. Compared with participants in the first quartile group, those in the fourth quartile group had 8.54% (6.60, 10.51) and 5.98% (3.79, 8.22) increased eGFR for LIPA and MVPA, respectively. In contrast, individuals in the fourth quartile of total ST were associated with a 5.42% (3.61, 7.19) decrease of eGFR compared to those in the first quartile group.

**Table 2:** Associations of physical activity and sedentary time with estimated glomerular filtration rate.

Sedentary behavior	Model 1		Model 2		Model 3	
	Difference (95% CI)	*p*	Difference (95% CI)	*p*	Difference (95% CI)	*p*
Low-intensity physical activity						
Quartile 1	Ref		Ref		Ref	
Quartile 2	10.58% (8.29, 12.91)	<0.001	4.91% (3.09, 6.76)	<0.001	4.89% (3.07, 6.74)	<0.001
Quartile 3	15.81% (13.42, 18.25)	<0.001	6.83% (4.95, 8.74)	<0.001	6.82% (4.93, 8.73)	<0.001
Quartile 4	20.01% (17.53, 22.54)	<0.001	8.57% (6.63, 10.55)	<0.001	8.54% (6.60, 10.51)	<0.001
*p* for trend		<0.001		<0.001		<0.001
Moderate to vigorous physical activity						
Quartile 1	Ref		Ref		Ref	
Quartile 2	18.05% (15.70, 20.45)	<0.001	4.20% (2.29, 6.15)	<0.001	4.31% (2.39, 6.27)	<0.001
Quartile 3	23.83% (21.36, 26.34)	<0.001	4.22% (2.21, 6.27)	<0.001	4.39% (2.36, 6.45)	<0.001
Quartile 4	32.29% (29.65, 34.98)	<0.001	5.82% (3.64, 8.04)	<0.001	5.98% (3.79, 8.22)	<0.001
*p* for trend		<0.001		<0.001		<0.001
Sedentary time						
Quartile 1	Ref		Ref		Ref	
Quartile 2	−6.79% (−8.70, −4.84)	<0.001	−0.97% (−2.71,0.81)	0.283	−0.95% (−2.69, 0.82)	0.292
Quartile 3	−13.63% (−15.40, −11.83)	<0.001	−4.37% (−6.09, −2.62)	<0.001	−4.39% (−6.11, −2.64)	<0.001
Quartile 4	−18.69% (−20.36, −17.00)	<0.001	−5.31% (−7.08, −3.50)	<0.001	−5.42% (−7.19, −3.61)	<0.001
*p* for trend		<0.001		<0.001		<0.001

Model 1: crude model; Model 2: adjusted for age, sex, educational level, ethnicity, and BMI; Model 3: further adjusted for smoking, alcohol use, and ratio of family income to poverty.

For the associations of PA, ST, and CKD prevalence (**Table 3**), compared with the participants in the first quartile, those in the fourth quartile group had an associated odds of 0.48 (0.35, 0.65), 0.58 (0.38, 0.86), and 1.64 (1.17, 2.31) for LIPA, MVPA, and total ST, respectively.

**Table 3:** Associations of physical activity and sedentary time with odds of CKD.

Sedentary behavior	Model 1		Model 2		Model 3	
	OR (95% CI)	*p*	OR (95% CI)	*p*	OR (95% CI)	*p*
Low-intensity physical activity						
Quartile 1	Ref		Ref		Ref	
Quartile 2	0.51 (0.41, 0.63)	<0.001	0.77 (0.60, 0.98)	<0.001	0.76 (0.61, 0.97)	<0.001
Quartile 3	0.29 (0.23, 0.37)	<0.001	0.55 (0.42, 0.73)	<0.001	0.55 (0.41, 0.72)	<0.001
Quartile 4	0.23 (0.17, 0.30)	<0.001	0.49 (0.36, 0.66)	<0.001	0.48 (0.35, 0.65)	<0.001
*p* for trend		<0.001		<0.001		<0.001
Moderate to vigorous physical activity						
Quartile 1	Ref		Ref		Ref	
Quartile 2	0.33 (0.27, 0.41)	<0.001	0.90 (0.71, 1.14)	0.392	0.89 (0.70, 1.13)	0.355
Quartile 3	0.12 (0.09, 0.16)	<0.001	0.55 (0.39, 0.75)	<0.001	0.54 (0.39, 0.74)	<0.001
Quartile 4	0.07 (0.05, 0.10)	<0.001	0.57 (0.38, 0.85)	0.007	0.58 (0.38, 0.86)	0.009
*p* for trend		<0.001		<0.001		<0.001
Sedentary time						
Quartile 1	Ref		Ref		Ref	
Quartile 2	1.58 (1.13, 2.23)	0.009	0.89 (0.62, 1.29)	0.538	0.89 (0.62, 1.29)	0.543
Quartile 3	3.63 (2.69, 4.97)	<0.001	1.49 (1.06, 2.10)	0.021	1.51 (1.08, 2.13)	0.018
Quartile 4	5.61 (4.21, 7.60)	<0.001	1.60 (1.15, 2.26)	0.006	1.64 (1.17, 2.31)	0.005
*p* for trend		<0.001		<0.001		<0.001

Model 1: crude model; Model 2: adjusted for age, sex, educational level, ethnicity, and BMI; Model 3: further adjusted for smoking, alcohol use, and ratio of family income to poverty. CKD, chronic kidney disease.

Results of ISM are shown in **Tables 4**, **5**. Substituting 30 min/day of ST with equivalent LIPA/MVPA was associated with 1.19% (0.90,1.48) and 0.38% (0.06,0.70) increased of eGFR, respectively (**Table 4**). Substituting 30 min/day of ST with equivalent LIPA was associated with reduction in CKD, with an 11% (6%,15%) risk reduction. To note, replacing 30 min/day MVPA with equivalent LIPA was a potential benefit for renal function and CKD (**Table 5**), with a 1.26% (0.98, 1.55) increase of eGFR and a lower risk of CKD [OR: 0.89 (0.85,0.93)].

**Table 4:** Isotemporal substitution associations of ST, LIPA, and MVPA (per 30 min) with renal function.

Model ^a^	Sedentary time		Low-intensity PA		Moderate to vigorous PA	
	Difference (95% CI)	*p*	Difference (95% CI)	*p*	Difference (95% CI)	*p*
Single-factor model ^b^	−0.41% (**-**0.54, **-**0.28)	<0.001	1.35% (1.08, 1.62)	<0.001	0.70% (0.41, 1.00)	<0.001
2-Factor model ^c^	N/A		1.19% (0.90,1.48)	<0.001	0.38% (0.06, 0.70)	0.021
2-Factor model ^c^	−0.21% (−0.34, −0.06)	0.003	N/A		0.29% (−0.01, 0.59)	0.060
2-Factor model ^c^	−0.33% (−0.47, −0.19)	<0.001	1.26% (0.98, 1.55)	<0.001	N/A	
Partition model ^d^	−0.18% (−0.33, −0.04)	0.015	1.17% (0.88, 1.47)	<0.001	−1.88% (−0.18, 0.47)	0.399

a All variables expressed in 30-min units. All models adjusted for age, sex, educational level, ethnicity, smoking, alcohol use, ratio of family income to poverty, BMI, hypertension, diabetes, and cardiovascular disease.

b Single-factor models are results from separate models for each activity variable (sedentary time, LIPA, and MVPA), adjusted for covariates.

c Two-factor model results are from separate models that included either ST and LIPA, or ST and MVPA, or LIPA and MVPA, and adjusted for covariates, “N/A” indicates that the variable was not included in the model.

d Partition model shows results from a single model that includes sedentary time, LIPA, MVPA, and covariates.

**Table 5:** Isotemporal substitution associations of ST, LIPA, and MVPA (per 30 min) with CKD.

Model^a^	Sedentary time		Low-intensity PA		Moderate to vigorous PA	
	HR (95% CI)	*p*	HR (95% CI)	*p*	HR (95% CI)	*p*
Single-factor model^b^	1.03 (1.01, 1.05)	0.003	0.89 (0.85, 0.93)	<0.001	0.91 (0.85, 0.97)	0.007
2-Factor model^c^	N/A		0.89 (0.85,0.94)	<0.001	0.93 (0.87, 1.00)	0.065
2-Factor model^c^	1.01 (0.99,1.03)	0.297	N/A		0.98 (0.91,1.04)	0.446
2-Factor model^c^	1.02 (1.00,1.04)	0.051	0.89 (0.85,0.93)	<0.001	N/A	
**Partition model** ^d^	1.01 (0.99, 1.03)	0.393	0.90 (0.86, 0.94)	<0.001	0.98 (0.92, 1.05)	0.619

a All variables expressed in 30-min units. All models adjusted for age, sex, educational level, ethnicity, smoking, alcohol use, ratio of family income to poverty, BMI, hypertension, diabetes, and cardiovascular disease.

b Single-factor models are results from separate models for each activity variable (sedentary time, LIPA, and MVPA), adjusted for covariates.

c Two-factor model results are from separate models that included either ST and LIPA, or ST and MVPA, or LIPA and MVPA, and adjusted for covariates, “N/A” indicates that the variable was not included in the model.

d Partition model shows results from a single model that includes sedentary time, LIPA, MVPA, and covariates.

**Figure 2:**
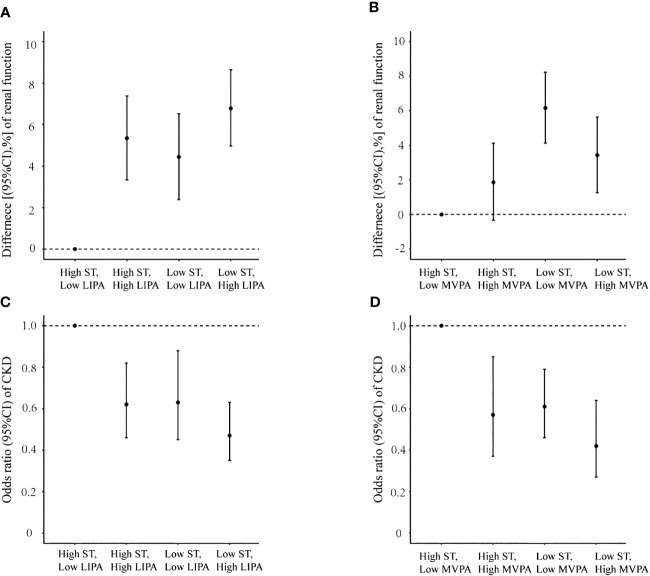
Joint association of sedentary time and physical activity with renal function and chronic kidney disease prevalence. **(A)** Sedentary time and low-intensity physical activity with renal function; **(B)** sedentary time and moderate to vigorous physical activity with renal function; **(C)** sedentary time and low-intensity physical activity with chronic kidney disease prevalence; **(D)** sedentary time and moderate to vigorous physical activity with chronic kidney disease prevalence.

Results of the joint analysis are presented in **Figure 2**. Significantly lower eGFR was observed for participants with both “high ST–low LIPA” than for those with “low ST–high LIPA”. Moreover, eGFR in participants with “high ST–low MVPA” was lower significantly than in participants with “low ST–high MVPA”. Consistently, the highest CKD risk was observed for both participants with high ST–low LIPA and those with high ST–low MVPA.

Subgroup analyses generally yielded similar results, although some differences did not reach statistical significance (**Supplementary Tables S1**–**S3**). Statistically significant interactions were observed for some factors, including sex/age/BMI/ethnicity for the relationship between LIPA and eGFR, age/BMI for the relationship between MVPA and eGFR, BMI/ethnicity for the relationship between total ST and eGFR, BMI/ethnicity for the relationship between LIPA and CKD, and BMI for the relationship between MVPA and CKD.

## Discussion

Using the NHANES data, this is the largest study to evaluate the relationships of accelerometer-measured PA and ST with eGFR/CKD in a representative sample of U.S. adults. We found that higher LIPA and MVPA were each associated with significantly higher levels of eGFR and lower likelihood of CKD. In contrast, more ST was associated with lower eGFR and higher odds of CKD. Specifically, substituting 30 min/day of ST/MVPA with equivalent LIPA could be associated with a lower risk of CKD. These findings suggest that both PA and sedentary behavior may be contributing factors affecting renal function and CKD and replacing sedentary behavior/MVPA with LIPA may benefit CKD in U.S. adults.

Previous studies have also investigated the possible associations between PA and renal function/CKD, with inconsistent results. Two cross-sectional studies of older adults, based on accelerometer-measured data, suggest that sedentary behavior (detrimentally) and PA (beneficially) may affect renal function (28, 34). Several cohort studies reported that higher PA was associated with a smaller decrease in eGFR or/and with a decreased risk of incident CKD (24, 35–37). Three recent meta-analyses evaluated the beneficial association between PA and a lower risk of CKD (38–40). In contrast, both a population-based longitudinal Doetinchem study and a prospective cohort study indicate that no association was observed with PA (based on self-reported measures) with eGFR decline (16, 17).The inconsistency might be imputed to many factors, including study design, information bias based on self-report, and different targeted population. We believe that our study based on a large population and accelerometer-measured data may provide robust evidence.

Limited information is available regarding the association between ST and risk of CKD. The ADDITION-Leicester study (Anglo-Danish-Dutch Study of Intensive Treatment in People With Screen Detected Diabetes in Primary Care, Leicester arm) with 6,749 adults reported that lower levels of total sitting time were associated with lower prevalence of CKD [OR, 0.74 (95% CI, 0.62–0.92) for lowest vs. highest tertile] (23). The UK Biobank study with 329,758 participants also found that a longer television viewing time, a commonly used index for total STs, was positively associated with higher odds of CKD (14). However, both of these previous studies were based on self-reported ST, which may lead to underestimated relationships between sedentary behaviors and health risk (26, 27). The use of accelerometer-measured ST may reduce the potential measurement errors and some other bias, which is inherent in self-report data. To our knowledge, only one study has investigated the relationship between accelerometer-measured ST and CKD. This study, which recruited 1,268 participants in the Framingham Offspring Study (FOS), suggested that every 30-min/day increase in total ST was associated with 16% (95% CI, 4%–29%) higher odds of CKD (15). However, it might be difficult to compare our results directly with the FOS study because the FOS study was mainly targeted on older adults of white individuals of European ancestry. Moreover, the studies are different in participant inclusion/exclusion criteria, sample sizes, and covariates. The findings of positive association between total ST and odds of CKD, identified in a study with a U.S. national, cross-sectional study of 5,990 adults, added evidence for the relationship of objectively measured sedentary behaviors with CKD.

Isotemporal substitution is an emerging technique that explores the relationship between PA, ST, and health due to the finite nature of time. Only two studies in Japan have examined the potential impacts of replacing ST with PA on renal function/CKD, using an isotemporal substitution model. A cross-sectional study with 174 older Japanese adults suggested that replacement of 30 min/day of ST with an equivalent time of MVPA was beneficially associated with eGFR (β = 5.49, *p* < 0.05) and replacement with an equivalent LIPA time was not significantly associated with eGFR (β = 2.26, *p* = 0.112) (28). A study using the data from a Japan Multi-Institutional Collaborative Cohort Study reported that replacing 1 h of sedentary behavior with 1 h of PA was associated with approximately 3% to 4% lower OR of CKD (41). To our knowledge, our study is the first to examine the associations of accelerometer-measured ST and PA with renal function/CKD using isotemporal substitution modeling in U.S. adults. Our results are consistent with previous results, and it highlights that LIPA has a stronger protective effect on CKD compared to MVPA when ST was replaced by equivalent LIPA or MVPA. PA has been shown to reduce inflammation and oxidative stress in patients with CKD (41). However, excessive PA was not traditionally recommended because of the possibility of impairing renal function and increasing proteinuria, which may account for our study results. Another possible reason is that patients with renal impairment may have a lifestyle change like physical activity increasement. According to KDIGO 2022 Clinical Practice Guideline for Diabetes Management in Chronic Kidney Disease, patients with CKD are advised to undertake moderate-intensity physical activity for a cumulative duration of at least 150 min per week. Hence, individuals with renal impairment or CKD may change their unhealthy lifestyle, like from lower level of physical activity to higher level of physical activity. However, the cross-sectional design of this study made it difficult to consider the potential impact if changes of these lifestyle factors. Therefore, further research should focus on the dose of PA, such as frequency, duration, and intensity.

In subgroup analyses, we observed a possible effect modification by BMI, showing stronger associations between LIPA and eGFR, as well as CKD, in overweight/obese individuals (BMI ≥ 25 kg/m^2^), compared with others (BMI < 25 kg/m^2^). This result was consistent with findings from a previous study suggesting that obese individuals were more sensitive to improved renal function with LIPA intervention (42). The possible mechanism for the effect modification by BMI may be partly related to the mitigation of insulin resistance, inflammation and oxidative stress following weight loss. From the perspective of public health, individuals should be encouraged to increase physical activity to maximize the kidney health benefits, especially in obesity. Furthermore, significant interaction was observed in the subgroups of age, sex, and ethnicity, which provide evidence that the clinical manager should consider the flexible PA intervention target on different populations in nephropathy management.

Our study has a series of strengths. First, we used data from a nationally representative sample of U.S. adults, which makes it possible for us to generalize our findings. Second, the information on sedentary behavior was objectively collected by using accelerometry. Third, the relatively large sample size makes it possible to provide more stable and precise estimates and allows us to conduct several subgroup analyses. Fourth, the comprehensive data collection of NHANES allowed us to consider the potential effects of a wide range of confounding factors.

Several limitations should also be acknowledged. First, the direction of causality between sedentary behaviors and CKD cannot be determined due to the cross-sectional design of this study. Second, we used only one single measurement of eGFR less than 60 mL/min/1.73 m^2^ to diagnose CKD. In clinical settings, it requires two measurements of eGFR taken at least 90 days apart to diagnose CKD. As a result, our study may have included some cases with acute CKD, which may dilute the relationships. Third, we did not include visceral obesity, dyslipidemia, and diet as covariables, which are important risk factors for CKD. Fourth, as the accelerometers would be taken off during swimming, individuals participating in such activity may have been misclassified in more sedentary quartiles or lower physical activity quartiles in our analysis. However, the bias would attenuate our findings of a strong association between ST/PA and renal function/CKD. Finally, our study only focused on the density of PA and total ST; further studies are needed to evaluate the cause-and-effect associations between various physical activities and sedentary pattern (total ST, sedentary duration, and sedentary breaks) and renal function/CKD.

## Conclusion

In conclusion, we found that LIPA and MVPA were each associated with an increase in eGFR and lower odds of CKD in this nationally representative U.S. adult population. In contrast, more total ST was associated with lower eGFR and higher odds of CKD. Intervention efforts to reduce total ST and increase LIPA/MVPA might be important for preventing CKD in adults.

## Data availability statement

The original contributions presented in the study are included in the article/**Supplementary Material**. Further inquiries can be directed to the corresponding authors.

## Ethics statement

The studies involving humans were approved by the NCHS Research Ethics Review Board. The studies were conducted in accordance with the local legislation and institutional requirements. The participants provided their written informed consent to participate in this study.

## Author contributions

XS: Writing – review & editing, Writing – original draft, Validation. YL: Writing – review & editing, Validation. AA: Writing – review & editing. QL: Writing – review & editing, Supervision, Project administration, Conceptualization. YB: Methodology, Formal Analysis, Data curation, Writing – review & editing, Supervision, Project administration, Conceptualization.
