# Supplementary material for: Associations of accelerometer-measured physical activity and sedentary time with renal function and chronic kidney disease: a national population-based study
Source: Front Endocrinol (Lausanne). 2024 Jun 17;15:1403998. doi: 10.3389/fendo.2024.1403998 (PMC11215116; doi:10.3389/fendo.2024.1403998)
Supplement: Supplementary file 1 [file Table_1.docx]

**Table S1. Association of low-intensity physical activity with estimated glomerular filtration rate and chronic kidney disease prevalence in stratified analysis**

|  | **Estimated glomerular filtration rate** | | **CKD** | |
| --- | --- | --- | --- | --- |
|  | **Difference (95%CI)** | ***P*** | **OR (95%CI)** | ***P*** |
| **Sex** |  | 0.004* |  | 0.380 |
| Male | 0.94% (0.55, 1.33) | <0.001 | 0.90 (0.84, 0.96) | 0.001 |
| Female | 1.57% (1.18, 1.96) | <0.001 | 0.87 (0.82, 0.92) | <0.001 |
| **Age** |  | <0.001* |  | 0.341 |
| <65 years | 0.95% (0.65, 1.25) | <0.001 | 0.85 (0.78, 0.92) | <0.001 |
| ≥65 years | 1.72% (1.13, 2.32) | <0.001 | 0.89 (0.85, 0.94) | <0.001 |
| **BMI** |  | <0.001* |  | 0.046* |
| <25 kg/m^2^ | 0.85% (0.37, 1.33) | <0.001 | 0.95 (0.87, 1.02) | 0.158 |
| ≥25 kg/m^2^ | 1.58% (1.25, 1.91) | <0.001 | 0.86 (0.82, 0.91) | <0.001 |
| **Ethnicity** |  | 0.019* |  | 0.013* |
| White | 1.11% (0.76, 1.47) | <0.001 | 0.93 (0.88, 0.97) | 0.003 |
| Black | 1.59% (0.88, 2.30) | <0.001 | 0.80 (0.71, 0.90) | <0.001 |
| Others | 1.69% (1.18, 2.21) | <0.001 | 0.81 (0.73, 0.90) | <0.001 |

Abbreviations: CKD, chronic kidney disease; BMI, body mass index.

^a^Model was adjusted for age, sex (not in sex-stratified analysis), educational level (not in education-stratified analysis), ethnicity (not in ethnicity-stratified analysis), BMI, smoking, alcohol use, and ratio of family income to poverty.

**Table S2. Association of moderate to vigorous physical activity with estimated glomerular filtration rate and chronic kidney disease prevalence in stratified analysis**

|  | **Estimated glomerular filtration rate** | | **CKD** | |
| --- | --- | --- | --- | --- |
|  | **Difference (95%CI)** | ***P*** | **OR (95%CI)** | ***P*** |
| **Sex** |  | 0.718 |  | 0.512 |
| Male | 1.02% (0.64, 1.40) | <0.001 | 0.86 (0.08, 0.95) | 0.003 |
| Female | 0.48% (0.02, 0.94) | 0.040 | 0.95 (0.86, 1.02) | 0.217 |
| **Age** |  | <0.001* |  | 0.129 |
| <65 years | 0.62% (0.32, 0.91) | <0.001 | 0.82 (0.73, 0.92) | 0.001 |
| ≥65 years | 0.70% (-0.13, 1.54) | 0.100 | 0.94 (0.87, 1.02) | 0.157 |
| **BMI** |  | 0.001* |  | 0.029* |
| <25 kg/m^2^ | 0.63% (0.17, 1.08) | 0.007 | 0.86 (0.75, 0.99) | 0.037 |
| ≥25 kg/m^2^ | 0.86% (0.47, 1.24) | <0.001 | 0.92 (0.85, 0.99) | 0.039 |
| **Ethnicity** |  | 0.882 |  | 0.288 |
| White | 0.51% (0.14, 0.88) | <0.001 | 0.95 (0.88, 1.02) | 0.190 |
| Black | 1.40% (0.50, 2.32) | 0.002 | 0.80 (0.64, 0.98) | 0.040 |
| Others | 0.77% (0.26, 1.28) | 0.003 | 0.80 (0.68, 0.94) | 0.008 |

Abbreviations: CKD, chronic kidney disease; BMI, body mass index.

^a^Model was adjusted for age, sex (not in sex-stratified analysis), educational level (not in education-stratified analysis), ethnicity (not in ethnicity-stratified analysis), BMI, smoking, alcohol use, and ratio of family income to poverty

**Table S3. Association of sedentary time with estimated glomerular filtration rate and chronic kidney disease prevalence in stratified analysis**

|  | **Estimated glomerular filtration rate** | | **CKD** | |
| --- | --- | --- | --- | --- |
|  | **Difference (95%CI)** | ***P*** | **OR (95%CI)** | ***P*** |
| **Sex** |  | 0.103 |  | 0.253 |
| Male | -0.42% (-0.58, -0.25) | <0.001 | 1.04(1.02, 1.07) | 0.002 |
| Female | -0.45% (-0.65, -0.25) | <0.001 | 1.02 (0.99, 1.04) | 0.246 |
| **Age** |  | 0.297 |  | 0.265 |
| <65 years | -0.38% (-0.52, -0.23) | <0.001 | 1.04(1.00, 1.08) | 0.038 |
| ≥65 years | -0.31% (-0.58, -0.05) | 0.021 | 1.03(1.01, 1.05) | 0.010 |
| **BMI** |  | 0.001* |  | 0.332 |
| <25 kg/m^2^ | -0.33% (-0.56, -0.10) | 0.005 | 1.03 (0.99, 1.06) | 0.100 |
| ≥25 kg/m^2^ | -0.46% (-0.62, -0.31) | <0.001 | 1.03(1.01, 1.05) | 0.011 |
| **Ethnicity** |  | 0.020* |  | 0.232 |
| White | -0.32% (-0.49, -0.15) | <0.001 | 1.02(1.00, 1.04) | 0.090 |
| Black | -0.37% (-0.68, -0.05) | 0.022 | 1.03(0.99, 1.08) | 0.134 |
| Others | -0.68% (-0.94, -0.43) | <0.001 | 1.05(1.00, 1.09) | 0.033 |

Abbreviations: CKD, chronic kidney disease; BMI, body mass index.

^a^Model was adjusted for age, sex (not in sex-stratified analysis), educational level (not in education-stratified analysis), ethnicity (not in ethnicity-stratified analysis), BMI, smoking, alcohol use, and ratio of family income to poverty
